# Moderating effect of urban endowment factors on environmental regulations-productivity relationship in Beijing-Tianjin-Hebei region

**DOI:** 10.1016/j.heliyon.2023.e21252

**Published:** 2023-10-27

**Authors:** Mingyang Wang, Rong Gao, Hua Ma

**Affiliations:** College of Chemical Engineering and Environment, China University of Petroleum, Beijing, 102249, China

**Keywords:** Environmental regulation, Total factor productivity (TFP), Urban endowment factors, Moderating effect

## Abstract

In this paper, six major cities in the Beijing-Tianjin-Hebei region of China are classified into two groups, namely core city (Beijing) and industrial cities. The objective is to analyze the moderating effect of urban endowments in different urban contexts on the environmental regulation-productivity relationship using an econometric model. The results are: As environmental regulation intensifies, production in Beijing's high-polluting industries rises after falling, showing “innovation compensation” and “inefficient exit” effects. In contrast, the high-polluting industries in the industrial cities exhibit a “compliance cost” effect. In Beijing, four urban endowment factors, including economic development, technological innovation, human capital, and government intervention, can provide supportive conditions for business development under environmental regulatory pressure, primarily by reducing the “compliance cost” effect and enhancing the “innovation compensation” effect. In industrial cities, on the other hand, urban endowment factors have not had a positive moderating effect, and government intervention has even had a negative effect. We argue that government intervention may be the more fundamental urban endowment factor, which may affect the moderating outcomes of other endowment factors. Based on these findings, we propose that governments should make greater use of guiding and incentive-based environmental policy instruments, while reducing administrative interventions. Appropriate policy instruments can activate the positive moderating role of urban endowments and thus provide a better supportive environment for firms' technological innovation.

## 1 Introduction

Since the reform and opening up, China's economic take-off has been accompanied by environmental pollution and resource depletion brought about by the extensive economic growth mode, which has seriously affected the sustainability of development. As a result, China's development philosophy has begun to change. The introduction of “green development” (2012), “high-quality development” and “enhancing total factor productivity” (2017) signifies that the focus of the Chinese government on economic development has changed from “development scale” to “development quality and efficiency”. At the same time, China has made significant revisions to many critical environmental regulations, and the rigidity and intensity of environmental regulations have been significantly enhanced. Particularly in the field of air pollution prevention and control, for example, during the Tenth Five-Year Plan period (2001–2005), China began setting total control targets for crucial air pollutants; in 2013, the State Council issued the Action Plan for Air Pollution Prevention and Control to control haze pollution; in 2018, significant amendments were made to the Air Pollution Prevention and Control Act. China's large regional development gaps often lead to very different results of environmental policy implementation. For example, in cities with large development gaps, the same type and intensity of environmental regulation may have the opposite result of promoting or inhibiting economic efficiency. To clarify heterogeneity mechanism in the economic impacts of environmental regulations, the following research questions have been developed to guide the study: What kind of urban heterogeneity does the economic impact of environmental regulations exhibit? What is the mechanistic role of urban endowment factors in this urban heterogeneity?

Beijing-Tianjin-Hebei, as a key area for China's economic development and air pollution control, is also a region with large differences in regional development. Since the State Council promulgated the Air Pollution Prevention and Control Act in 2013, the Beijing-Tianjin-Hebei region has successively introduced a series of air pollution prevention and control policies, which will certainly have a complex impact on the economic system of the region. Therefore, using the region as a case area, this paper analyzes the moderating role of urban endowment factors on the environmental regulation-productivity relationship and its heterogeneous mechanisms.

## 2 Literature review and theoretical analysis

In recent years, theories on the productivity effects of environmental regulation have flourished. The “compliance cost” effect argues that strict environmental regulations require firms to increase input costs to meet compliance requirements [[1], [2], [3]]; the input of compliance costs will have a crowding-out effect on technological innovation activities, reducing the efficiency of enterprise resource allocation and thus reducing the total factor productivity (TFP) of enterprises [[4], [5], [6], [7]]. On the other hand, Porter and Linde argued that environmental regulation of appropriate intensity could facilitate technological innovation in firms [8]. The reduction and harmless treatment of pollutants [9] helps enterprises to save resources and energy [10], reduce production costs [11], enhance the market share of green products and the competitiveness of enterprises [12,13], and generate an “innovation compensation” effect that wholly or partially offsets the costs of environmental regulation [14]. Some researchers have confirmed Porter's hypothesis through empirical studies [[15], [16], [17], [18]]. Some other researchers have constructed mediation effect models to confirm that environmental regulation can promote green technological innovation in firms, thereby increasing their TFP [[19], [20], [21]]. Environmental regulation can not only increase R&D investment and promote enterprises to upgrade technology and equipment [22] but also improve TFP by improving energy efficiency [23], optimizing resource allocation [21,24], improving management level [25], and promoting industrial structure upgrading [26]. Some scholars also argue that the “innovation compensation” effect of environmental regulation can only partially offset the cost of compliance [[27], [28], [29], [30]]; TFP is promoted when the “innovation compensation” effect of environmental regulation is greater than the “compliance cost” effect and is inhibited when the opposite is true [[31], [32], [33], [34]]. Under the influence of different contextual factors (e.g., different urban endowment factors), these two “effects”, which act in opposite directions, may be promoted or suppressed to different degrees, and the superposition of the two effects thus presents a heterogeneous outcome of environmental regulation.

In the study of the heterogeneity mechanism, the current academic community has mainly focused on the intermediary effect, while the study of the moderating effect has been largely neglected. The intermediary effect refers to the effect of the independent variable affecting the dependent variable through the mediating factor, and the moderating effect refers to the effect of the moderating factor as a contextual factor supporting or weakening the influence of the independent variable on the dependent variable; compared with the former, the latter effect is more fundamental but often neglected due to being more implicit. This study attempts to analyze the moderating effect of urban endowment factors on the environmental regulations-productivity relationship and its heterogeneous impact.

The urban endowment is a collection of various elements unique to cities formed under particular historical backgrounds, natural conditions, and functional positioning, including natural factors such as geographical location, resources, and climate, and development factors such as economy, technology, human resources, and management level, which are the objective basis for regional economic development [[35], [36], [37]]; among them, the development factors may not only act directly on economic development, but may also have an indirect impact on economic transformation through moderating effects. The direct impact of urban endowment factors such as economic base, technological innovation, human capital and government intervention on productivity has been well studied. Researchers have argued that high levels of economic development have a strong ability to apply technological innovation and thus can improve TFP [6]. Technological progress and human capital can provide knowledge and specialized experience for regional development [38], improve production R&D and management of enterprises [6], reduce production costs, and improve production efficiency [10]. The government can intervene in the market through financial subsidies and tax breaks [39] to compensate for market failures, reduce enterprise R&D risks, and stimulate innovative activities [40], thus enhancing TFP [41]. In contrast, imperfect policy interventions can further distort market outcomes by leading to resource misallocation and reduced efficiency [42]. However, to the best of our knowledge, there is a lack of research on the moderating role of urban endowment factors on the relationship between environmental regulation and productivity. The findings of this paper will help to understand the moderating role of urban endowments and shed light on the mechanisms of heterogeneity in the impact of environmental regulation.

## 3 Research methodology and data

This paper uses panel data from 2003 to 2017 for six major cities in the Beijing-Tianjin-Hebei region of China as a sample. And the data are obtained from the China City Statistical Yearbook, China Industrial Statistical Yearbook, China Environmental Statistical Yearbook, Hebei Economic Yearbook, Statistical Yearbooks and Statistical Bulletins of each city.

### 3.1 Econometric models

Econometric models are often used to test economic theory and policy effects. In this paper, an empirical analysis is constructed using econometric models based on urban panel data. The objective is to test the theoretical relationships between urban endowment factors, environmental regulations and productivity and to explore the mechanisms behind these relationships.

Firstly, we construct an econometric model with TFP, which characterizes the economic performance of the industry, as the explained variable and environmental regulation as the explanatory variable (see equation (1)). Specifically, we use a fixed effects model to eliminate error terms that vary over time but not with individuals. And to ensure the validity of the regression results, we performed a unit root test on the panel data.

(1) $$\ln\;{TFP}_{it}=\beta_{0}+\sum_{a=1}^{3}\beta_{a}{envreg}_{it}^{a}+\sum {endow}_{it}+\sum {control}_{it}+\delta_{t}+\varepsilon_{it}$$

where $\ln\;{TFP}_{it}$ denotes the logarithm of TFP in year *t* for city *i*; ${envreg}_{it}^{a}$ denotes the primary, secondary, and tertiary terms of environmental regulation intensity; ${endow}_{it}$ denotes city endowment variables, including economic development (lnGDPpc), technological innovation (lnR&D), human capital (humcap), and government intervention (gov); ${control}_{it}$ denotes control variables, including foreign direct investment (FDI) and industrial structure (indstrc); $\delta_{t}$ is the fixed effect; $\varepsilon_{it}$ is the random error term.

To further test the moderating effect of urban endowment factors on the impact of environmental regulation on TFP, equation (2) is constructed by adding the interaction term between endowment variables and environmental regulation on the basis of equation (1).

(2) $$\ln\;{TFP}_{it}=\beta_{0}+\beta_{1}{envreg}_{it}+\beta_{2}{envreg}_{it}{\times endow}_{it}+\sum {endow}_{it}+\sum {control}_{it}+\delta_{t}+\varepsilon_{it}$$

where ${endow}_{it}=\left\{lnGDPpc,lnR\&D,humcap,gov\right\}$, ${endow}_{it}\times {envreg}_{it}$ is the interaction term between urban endowment and environmental regulation, and both sides of the model are biased against environmental regulation to obtain $\frac{\partial \ln\;{TFP}_{it}}{\partial {envreg}_{it}}=\beta_{1}+\beta_{2}{endow}_{it}$, $\beta_{2}$ measures the moderating effect with a positive (negative) sign, indicating a positive (negative) moderating effect of urban endowment factors on the relationship between environmental regulation and TFP.

### 3.2 Variables and data description

#### 3.2.1 TFP

Total factor productivity (TFP) is a reference indicator to measure economic efficiency by using the ratio of the total output of production units to total input factors in a certain period. It indicates the portion of output increase caused by factors other than production factors, such as technological progress, when the number of inputs of production factors such as capital, labor, and land remains unchanged [43,44]. This paper employs the Data Envelopment Analysis (DEA) method [45] to estimate the industrial TFP of the case city using a hybrid radial EBM model [46,47] combined with a global covariate MI model with capital stock and labor force as inputs [[48], [49], [50]] and industrial value added as an output [51]. Among them, capital stock is calculated using the perpetual inventory method [52] with the net fixed assets of industrial enterprises above the scale in 2003 as the initial capital stock; labor input is the number of employees in industrial enterprises above the scale; and industrial value added is calculated using the actual value of industrial enterprises above the scale deflated by 2003 as the base period.

The Malmquist Index (MI) indicates the change in production efficiency: the distance between the input-output mix and the production frontier surface. In this paper, the MI index is calculated using the global reference, i.e., the frontier constructed jointly for all periods as the reference frontier [53], and MI > 1 (<1) indicates an increase (decrease) in total factor productivity.

#### 3.2.2 Environmental regulation

Currently, academics measure environmental regulation by several types of indicators, including pollution control cost expenditure [[54], [55], [56], [57]], pollutant emission or removal level [[58], [59], [60], [61]], and informal environmental regulation type [[62], [63], [64]]. Among them, pollution removal rate represents the direct behavioral response of industrial industries under the current environmental regulation, which can reflect the intensity of regulation and its changes sensitively. At the same time, this type of data is more available and comparable. Therefore, in this paper, two indicators, sulfur dioxide removal rate, and soot removal rate [65], are selected to construct a comprehensive index of atmospheric environmental regulation intensity using the integrated index method [66].

#### 3.2.3 Urban endowment variables and control variables

Four urban endowment variables, namely economic development, technological innovation, human capital and government intervention, are used to analyze the moderating effects of urban endowment factors. In addition, in order to control the omitted variable error, foreign direct investment (FDI) and industrial structure (indstrc), which have an impact on economic scale and growth rate, are selected as control variables. The measures of urban endowment variables and control variables are shown in Table 1.

**Table 1:** Measures of urban endowment variables and control variables.

Variable Type	Variable Name	Symbol	Measurements
urban endowments	economic development	GDPpc	GDP per capita based on 2003 (10,000 CNY)
	technological innovation	R&D	Number of patents granted by cities (pieces)
	human capital	humcap	Number of highly skilled and educated personnel in scientific research, technical service, geological exploration, etc./number of employees in urban units at the end of the year (persons/100 persons)
	government intervention	gov	Government fiscal spending/GDP (CNY/100 CNY)
control variables	foreign direct investment	FDI	The actual use of foreign capital/GDP (CNY/100CNY)
	industrial structure	indstrc	Value added of tertiary industry/value added of secondary industry

#### 3.2.4 Pollution industry division

Academics generally classify the pollution characteristics of industries based on pollutant emissions or emission intensity [[67], [68], [69]]. This paper classifies 37 industrial sectors into high, medium, and low pollution categories based on the pollution intensity index (PI) [70] of each sector. Among them, the high-polluting industries (with a mean PI value of 0.178) include 12 sectors such as electric power and heat production, the medium-polluting industries (with a mean PI value of 0.018) include 13 sectors such as textile industry, and the low-polluting industries (with a mean PI value of 0.002) include 12 sectors such as furniture manufacturing.

## 4 Results and discussion

### 4.1 Dynamics of environmental regulation and economic development

A systematic cluster analysis of six major cities in the Beijing-Tianjin-Hebei region, namely Beijing, Tianjin, Shijiazhuang, Tangshan, Handan, and Baoding, was carried out using four indicators: the TFP index, the ratio of output value of the tertiary industry to that of the secondary industry, the energy consumption per unit of industrial added value, and the sulfur dioxide emission per unit of industrial added value. The clustering result is that Beijing itself is classified as one category, called “core city”, and the other five cities are clustered as “industrial cities”.

#### 4.1.1 Environmental regulation intensity dynamics

**Fig. 1:**
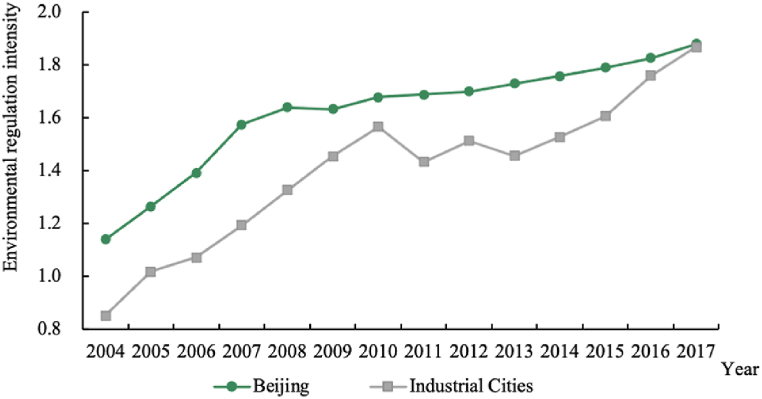
Changes in environmental regulation intensity, 2004–2017.

The changes in the environmental regulation intensity of the two types of cities from 2004 to 2017 are shown in Fig. 1.

As shown in Fig. 1, the environmental regulation intensity of both Beijing and industrial cities showed an overall upward trend during the study period, indicating that the air pollution prevention and control efforts in the region are continuously increasing. The intensity of environmental regulation in Beijing is significantly higher than that in industrial cities, while the regulatory intensity in industrial cities has also increased rapidly, eventually matching that of Beijing in 2017.

#### 4.1.2 Productivity dynamics

**Fig. 2:**
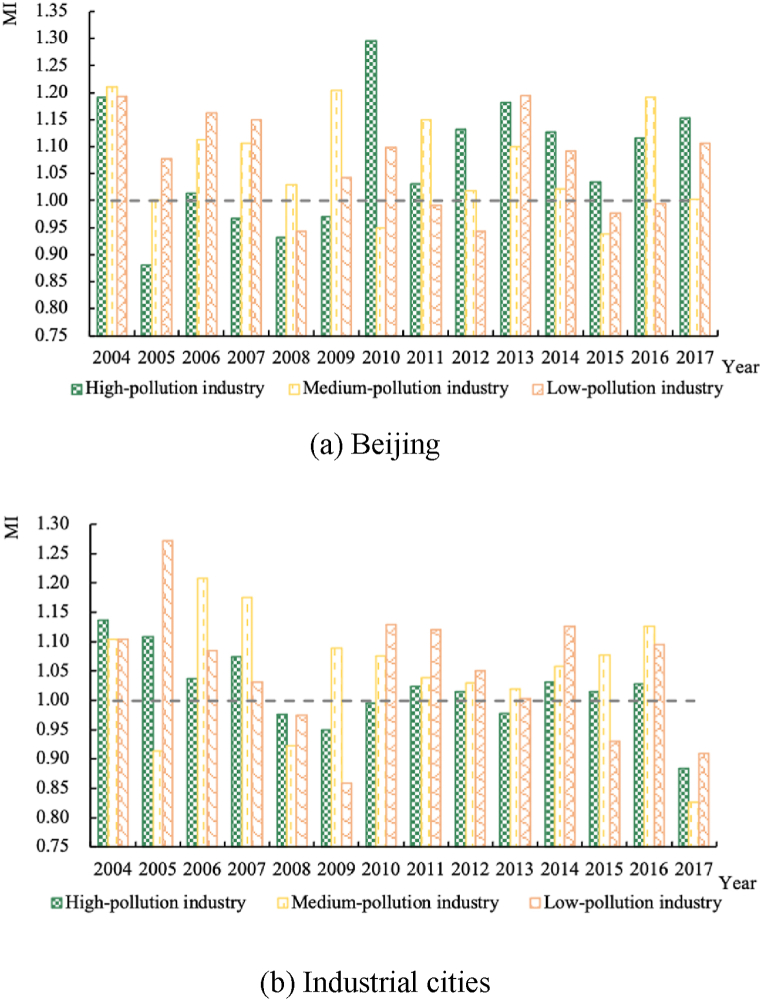
MI dynamics by polluting industries, 2004–2017.

The MI (the productivity change) dynamics of different pollution-intensive industries in the two types of cities from 2004 to 2017 are shown in Fig. 2.

As can be seen from Fig. 2(a), the MI of the high-polluting industry in Beijing was usually less than 1 before 2010 but greater than 1 after 2010, which indicates that the productivity of this industry was not suppressed by the increase in the intensity of environmental regulations. Possible reasons for this include: environmental regulations promote technological innovation, which increase TFP; and stringent environmental regulations force some highly polluting and inefficient firms to exit the market or move out of Beijing.

From Fig. 2(b), it can be seen that the TFP of high-polluting industries in industrial cities develops at a slower pace than that of other polluting industries, suggesting that environmental regulations may have had a greater dampening effect on the economic performance of high-polluting industries. And the productivity of all three types of industries declined significantly in 2017. This may be due to the fact that industrial cities did not make enough efforts in the first few years of the implementation of the Air Pollution Prevention and Control Action Plan (2013–2017), and in the last year, 2017, the pressure to meet the standards increased steeply, and the cost of compliance increased significantly, leading to a significant decline in production performance.

### 4.2 City and industry heterogeneity in the impact of environmental regulation on TFP

As differences in production processes and production efficiency of industries lead to different pollutant emission scales and treatment difficulties, there may be industry heterogeneity in the response of TFP under the same intensity of environmental regulations. The regression results of industry heterogeneity in the effects of environmental regulations on TFP in Beijing and industrial cities are shown in Table 2.

**Table 2:** Regression results of the relationship between the intensity of environmental regulation and TFP.

Variable Name	Dependent variable: lnTFP					
	Beijing			Industrial Cities		
	High-polluting industry	Medium-polluting industry	Low- polluting industry	High- polluting industry	Medium-polluting industry	Low- polluting industry
envreg	−8.6560*	−4.7723*	−1.3735	−3.4716	−8.3991***	−0.6101
	(4.0774)	(1.9198)	(5.8166)	(2.2033)	(1.8102)	(2.3423)
envreg^2^	3.4469*	1.145	1.0876	3.0708*	6.6152***	0.6426
	(1.4467)	(0.6712)	(2.3067)	(1.7993)	(1.4938)	(1.9563)
envreg^3^	–	–	–	−0.8702*	−1.6625***	−0.1899
	–	–	–	(0.4634)	(0.3918)	(0.5156)
lnGDPpc	−9.2588***	4.2804**	−4.9786	−0.1706	0.3906***	−0.3943**
	(2.0011)	(1.4323)	(4.07)	(0.1532)	(0.1336)	(0.1808)
LnR&D	2.8134***	−0.9558**	1.3783	0.0476	0.0465	0.3355**
	(0.4267)	(0.3504)	(0.927)	(0.1464)	(0.089)	(0.1315)
humcap	0.1614***	0.0201	0.0808	−0.1123**	0.2072***	−0.0476
	(0.0314)	(0.0216)	(0.0549)	(0.0428)	(0.0318)	(0.0378)
fdi	0.1238**	−0.0776**	0.0517	0.0165	−0.0275*	−0.014
	(0.0359)	(0.0209)	(0.0402)	(0.0183)	(0.014)	(0.0111)
gov	−0.1107***	0.0055	−0.0614	0.0111	−0.0325**	−0.0376**
	(0.0154)	(0.012)	(0.0417)	(0.0156)	(0.0125)	(0.0179)
lnis	1.7456**	−0.1041	0.9477	1.1308***	−0.0686	0.6273***
	(0.4484)	(0.2379)	(0.8689)	(0.1912)	(0.1685)	(0.1602)
Constant	−5.7374	6.2374**	−4.4216	1.8123	2.8183***	−0.7943
	(3.7968)	(2.2097)	(4.6044)	(1.1665)	(0.815)	(1.2503)
Sample	14	14	14	70	70	70
Adjust-R^2^	0.9834	0.9827	0.9276	0.3262	0.7699	0.5356
F	2234.1795***	29781.5682***	422.3273***	12.2196***	22.8876***	12.8601***

Note: The standard error is in parentheses; ***p < 0.01，**p < 0.05，*p < 0.10.

As can be seen from Table 2, the regression results are not significant for low-polluting industries in both types of cities. At the same time, environmental regulation has a significant impact on TFP for both high- and medium-polluting industries in both types of cities.

The relationship between regulation intensity and industry TFP in Beijing is as follows: a U-shaped curve for high-polluting industries, i.e., as the intensity of regulation increases, industry production decreases first and then rises; a monotonic decreasing curve for medium-polluting industries. The different curvilinear relationship between environmental regulation and productivity in high- and medium-polluting industries suggests that there are two possible regulatory impacts in high-polluting industries, namely, the “innovation compensation” effect and “inefficient enterprises exit” effect. Albrizio et al. studied manufacturing industries in OECD countries and found that an increase in the intensity of environmental regulations may lead to the closure of some low-productivity firms, thus raising the average productivity level of low-productivity industries [71].

The curves for high- and medium-polluting industries in industrial cities show inverted U and inverted N shapes, respectively, and the second half of the latter curve also shows an inverted U shape, suggesting that the output value of these two types of industries rises and then declines as regulation increases, which is the opposite of the trend for high-polluting industries in Beijing.

Table 3 shows the inflection points of TFP under increasing environmental regulations for different polluting industries in the two city categories. As shown in Table 3, for different city categories and polluting industry categories, the direction and degree of influence of environmental regulation on productivity show heterogeneity. The environmental regulation intensity at the inflection point of the curve for the high-polluting industry in Beijing is 1.26, and Beijing is now at the stage where the increase of environmental regulation intensity promotes the increase of TFP. At present, the environmental regulation levels of the five industrial cities have crossed the peak inflection point of the curve, i.e., the increase in the intensity of environmental regulation inhibits the increase in TFP. Among them, high-polluting industries reach the inflection point earlier than medium-polluting industries. This suggests at the same regulatory intensity, high-polluting industries are subject to greater actual regulatory pressure; as the intensity of urban environmental regulation increases, the compliance cost of high-polluting industries rises more rapidly, the “compliance cost” effect comes to the fore, and they enter the productivity suppression stage earlier; these findings are similar to those of Shen et al. [68].

**Table 3:** Inflection points of TFP under increasing environmental regulation intensity for different polluting industries.

City Types		Beijing	Industrial Cities	
Industry Types		High-polluting industry	High-polluting industry	Medium-polluting industry
Regulation intensity	Wave valley	1.26	–	1.05
	Wave peak	–	1.41	1.60

### 4.3 Moderating effect of urban endowment

Urban endowment is a collection of various natural and developmental factors, where developmental factors include hard power such as economic level, science and technology, and soft power factors such as human capital and government governance [72]. Different levels of urban endowments may affect the responsiveness of industrial TFP to environmental regulations. This paper uses the moderating effects model to analyze the moderating effects of urban endowment factors such as economic development, technological innovation, human capital, and government intervention on the environmental regulation-TFP relationship. The regression results of the models for different polluting industries in Beijing and industrial cities are shown in Table 4 and Table 5, respectively.

**Table 4:** Moderating effects of urban endowment factors on environmental regulation-TFP relationship in Beijing.

Variable Name	Dependent variable: lnTFP											
	economic development			technological innovation			human capital			government intervention		
	High-polluting industry	Medium- polluting industry	Low- polluting industry	High-polluting industry	Medium- polluting industry	Low- polluting industry	High-polluting industry	Medium- polluting industry	Low- polluting industry	High-polluting industry	Medium- polluting industry	Low- polluting industry
envreg	0.0369	−1.9058**	1.3794	−0.5501	−2.1198***	1.2136	0.6535	−1.6813**	1.5698	−0.1283	−1.9446**	1.4339
	(0.5783)	(0.5233)	(0.948)	(0.4786)	(0.4901)	(0.9453)	(0.6324)	(0.5891)	(1.03)	(0.4303)	(0.5015)	(0.887)
lnGDPpc	−8.2603***	4.5560**	−4.6369	−5.7962***	5.4560***	−3.9046	−8.7994***	4.2704**	−4.2166	−5.6031***	5.5009***	−3.9654
	(1.4485)	(1.35)	(3.4249)	(1.0356)	(1.2168)	(2.7771)	(1.6281)	(1.6313)	(3.7029)	(1.0289)	(1.3186)	(2.7843)
envreg*lnGDPpc	2.9618***	1.0821**	0.8878	–	–	–	–	–	–	–	–	–
	(0.7026)	(0.4077)	(1.5546)	–	–	–	–	–	–	–	–	–
lnR&D	2.3866***	−1.0982**	1.244	1.6851***	−1.3542***	1.0396	2.7964***	−0.9329*	1.2646	1.9939***	−1.2306**	1.1794
	(0.2983)	(0.3298)	(0.7819)	(0.2373)	(0.3182)	(0.7194)	(0.3869)	(0.4032)	(0.9146)	(0.2465)	(0.3463)	(0.727)
envreg*lnR&D	–	–	–	1.1767***	0.4295**	0.343	–	–	–	–	–	–
	–	–	–	(0.2275)	(0.1644)	(0.5767)	–	–	–	–	–	–
humcap	0.1326***	0.0114	0.0713	0.0996***	−0.0007	0.0615*	0.1490***	0.0191	0.065	0.1154***	0.0048	0.0649*
	(0.0138)	(0.0187)	(0.0367)	(0.0072)	(0.0181)	(0.0266)	(0.0223)	(0.0211)	(0.0421)	(0.0097)	(0.0197)	(0.0316)
envreg*humcap	–	–	–	–	–	–	0.2799**	0.1134*	0.0109	–	–	–
	–	–	–	–	–	–	(0.0717)	(0.0511)	(0.12)	–	–	–
gov	−0.0973***	0.009	−0.0567	−0.0859***	0.0132	−0.0532	−0.0818***	0.0142	−0.0489	−0.1771***	−0.0173	−0.0667
	(0.0104)	(0.012)	(0.034)	(0.0088)	(0.0121)	(0.0313)	(0.0172)	(0.0131)	(0.0321)	(0.0162)	(0.0206)	(0.0624)
envreg*gov	–	–	–	–	–	–	–	–	–	0.2940***	0.0995*	0.05
	–	–	–	–	–	–	–	–	–	(0.0312)	(0.0414)	(0.1247)
FDI	0.0846**	−0.0938***	0.0409	0.0631**	−0.1016***	0.0354	0.1556***	−0.0689**	0.0685	0.0686***	−0.0967***	0.0505
	(0.0237)	(0.0167)	(0.0473)	(0.0209)	(0.0174)	(0.0524)	(0.0167)	(0.0174)	(0.034)	(0.0103)	(0.0162)	(0.0374)
lnis	1.2896***	−0.2348	0.794	0.7717***	−0.4241**	0.6378*	1.0596***	−0.3031	0.6216	0.7950***	−0.4191**	0.6286
	(0.189)	(0.1662)	(0.5192)	(0.093)	(0.1388)	(0.3152)	(0.1627)	(0.1756)	(0.4231)	(0.0961)	(0.1565)	(0.3403)
Constant	−8.3257***	5.5176**	−5.3048	−4.7472**	6.8223***	−4.2966	−12.5227***	3.9853*	−6.5695	−7.2777***	5.7592**	−5.6737
	(1.4958)	(1.5292)	(3.4507)	(1.4961)	(1.6403)	(4.1081)	(1.6278)	(1.738)	(3.7101)	(0.9825)	(1.5473)	(3.0768)
Sample	14	14	14	14	14	14	14	14	14	14	14	14
Adjust-R^2^	0.9912	0.9852	0.9286	0.9931	0.9856	0.9287	0.9874	0.9857	0.9219	0.9944	0.9848	0.9243
F	6408.882***	14665.43***	431.05***	9661.179***	10715.64***	429.748***	1502.97***	4027.74***	447.417***	9164.388***	6875.184***	417.233***

Note: The standard error is in parentheses; ***p < 0.01，**p < 0.05，*p < 0.10.

**Table 5:** Moderating effects of urban endowment factors on the environmental regulation-TFP relationship in industrial cities.

Variable Name	Dependent variable: lnTFP											
	economic development			technological innovation			human capital			government intervention		
	High-polluting industry	Medium- polluting industry	Low- polluting industry	High-polluting industry	Medium- polluting industry	Low- polluting industry	High-polluting industry	Medium- polluting industry	Low- polluting industry	High-polluting industry	Medium- polluting industry	Low- polluting industry
envreg	−0.082	0.0543	−0.0566	−0.0731	0.2193	−0.0152	0.2224	0.0382	0.1605	−0.2506	0.2134	−0.1607
	(0.2581)	(0.2078)	(0.2039)	(0.2813)	(0.2016)	(0.2075)	(0.304)	(0.2285)	(0.1923)	(0.2609)	(0.1992)	(0.197)
lnGDPpc	−0.2828**	0.2718*	−0.3722**	−0.2808*	0.1635	−0.3758**	−0.4537***	0.2749*	−0.4805***	−0.1953	0.1891	−0.3120*
	(0.1231)	(0.1552)	(0.1564)	(0.1581)	(0.1664)	(0.1753)	(0.1666)	(0.161)	(0.1768)	(0.1428)	(0.1351)	(0.1695)
envreg*lnGDPpc	−0.0614	0.0629	−0.1782	–	–	–	–	–	–	–	–	–
	(0.1777)	(0.1813)	(0.1709)	–	–	–	–	–	–	–	–	–
lnR&D	0.1199	0.1276	0.3181***	0.12	0.1946*	0.3244**	0.203	0.1213	0.3816***	0.0696	0.1750*	0.2880**
	(0.1217)	(0.1054)	(0.1178)	(0.1393)	(0.112)	(0.1279)	(0.1299)	(0.0926)	(0.1188)	(0.1352)	(0.0957)	(0.1199)
envreg*lnR&D	–	–	–	−0.019	0.1132	−0.0467	–	–	–	–	–	–
	–	–	–	(0.0916)	(0.0837)	(0.0731)	–	–	–	–	–	–
humcap	−0.1353***	0.1822***	−0.0459	−0.1346***	0.1638***	−0.0454	−0.1714***	0.1814***	−0.0656*	−0.1192***	0.1669***	−0.0335
	(0.0392)	(0.0383)	(0.0346)	(0.0417)	(0.0407)	(0.0373)	(0.0453)	(0.0371)	(0.0369)	(0.0397)	(0.0346)	(0.0371)
envreg*humcap	–	–	–	–	–	–	0.1346	0.015	0.0461	–	–	–
	–	–	–	–	–	–	(0.0936)	(0.0961)	(0.0722)	–	–	–
gov	0.0012	−0.0388***	−0.0355**	0.0013	−0.0491***	−0.0358*	−0.0137	−0.0384***	−0.0453***	0.0131	−0.0502***	−0.0258
	(0.0139)	(0.0137)	(0.0164)	(0.0176)	(0.0152)	(0.0182)	(0.0161)	(0.0138)	(0.017)	(0.0174)	(0.0138)	(0.0182)
envreg*gov	–	–	–	–	–	–	–	–	–	−0.0416	0.04	−0.0454*
	–	–	–	–	–	–	–	–	–	(0.0294)	(0.0279)	(0.0239)
FDI	0.0257	−0.0222*	−0.0129	0.0252	−0.0167	−0.0139	0.0415*	−0.0211	−0.0062	0.0182	−0.0151	−0.0202*
	(0.018)	(0.0131)	(0.0109)	(0.0185)	(0.0131)	(0.0108)	(0.021)	(0.0165)	(0.0125)	(0.0183)	(0.0127)	(0.0114)
lnis	1.0088***	−0.2285	0.7410***	0.9816***	−0.3407**	0.6496***	0.9254***	−0.1748	0.5704***	1.0687***	−0.2825*	0.7058***
	(0.229)	(0.1831)	(0.2038)	(0.2071)	(0.1627)	(0.17)	(0.1576)	(0.1531)	(0.1386)	(0.1745)	(0.1561)	(0.1354)
Constant	0.3411	−1.0526*	−0.7095	0.313	−1.5893**	−0.8415	−0.3759	−0.9709**	−1.3196**	0.7572	−1.4432***	−0.5134
	(0.6482)	(0.602)	(0.597)	(0.8078)	(0.6081)	(0.6551)	(0.7102)	(0.4651)	(0.5424)	(0.7444)	(0.4744)	(0.5808)
Sample	70	70	70	70	70	70	70	70	70	70	70	70
Adjust-R^2^	0.3115	0.7356	0.5515	0.3107	0.7437	0.544	0.335	0.7351	0.5444	0.3309	0.746	0.5669
F	11.1761***	27.8165***	15.6889***	10.9184***	26.3335***	14.9533***	11.0359***	24.8454***	14.6349***	11.9884***	25.0602***	17.4594***

Note: The standard error is in parentheses; ***p < 0.01，**p < 0.05，*p < 0.10.

As shown in Table 4, all the four urban endowment factors in Beijing have a significant positive moderating effect on the relationship between environmental regulation and TFP for both high- and medium-polluting industries, that is, high levels of urban endowment factors can enhance the policy responsiveness of polluting industries when faced with high intensity environmental regulation, thus mitigating the negative impact of regulation and improving production efficiency. In addition, the higher the pollution intensity of the industry, the stronger the moderating and supporting effect of urban endowment factors.

The coefficients of the interaction term envreg*lnGDPpc in the models of high- and medium-polluting industries are 2.9618 and 1.0821, respectively, that is, for every 1 % increase in the level of economic development in Beijing, the negative effect of environmental regulation on TFP in high- and medium-polluting industries is reduced by 2.96 % and 1.08 %, respectively. Economic development in cities tends to generate more financial resources, including investment capital and government grants, which can be used by firms for green technology development activities [73]; in addition, larger and more diverse markets resulting from economic prosperity can provide firms with higher demand thus incentives to invest in innovation, which is particularly important for environmentally friendly innovation.

According to the coefficient of the interaction term envreg*lnR&D, for every 1 % increase in the level of technological innovation in Beijing, the negative effect of environmental regulation on TFP in high- and medium-polluting industries will be decreases by 1.18 % and 0.43 %, respectively. The clustering of related industries and innovative firms in cities creates a specialized ecosystem with a concentration of expertise, suppliers and potential partners that can accelerate green innovation through knowledge exchange, research collaboration and practice sharing.

According to the coefficient of the interaction term envreg*humcap, for every 1 ‰ increase in the level of human capital in Beijing, the negative effect of environmental regulation on TFP in high- and medium-polluting industries decreases by 2.8 % and 1.1 %, respectively, which suggests that high levels of human capital can improve the responsiveness of polluting industries to environmental regulation through their embedded knowledge and technological experience [42]; a diverse and highly educated labor force can bring in new ideas and problem-solving skills, and is a key enabler of research and development in areas such as energy efficiency, sustainable design and green solutions. In addition, improved environmental quality resulting from environmental regulation can further attract talents, thus enhancing the positive moderating effect of human capital.

According to the coefficients of the interaction term envreg*gov, for every 1 ‰ increase in the level of government intervention in Beijing, the negative effect of environmental regulation on the TFP of high- and medium-polluting industries decreases by 2.9 % and 1.0 %, respectively. These suggest that the government can promote the innovative behavior of polluting industries through guidance and incentives [40,41] such as financial subsidies, tax reductions and tax exemptions, and optimization of property rights systems, thus improving responsiveness to environmental regulations; the government can also improve the average productivity level of industries by raising regional access standards and prompting the outward relocation of inefficient polluting firms [55,74].

As shown in Tables 5 and in industrial cities, government intervention has a significant negative moderating effect on the relationship between environmental regulation and TFP in low-pollution industries, with the negative effect of environmental regulation on TFP increasing by 4.5 % for every 1 % increase in the level of government intervention. This suggests that the government intervention in these industrial cities may be inappropriate, lacking incentives for industrial technological innovation [75,76] on the one hand, and on the other hand, there may be the use of high-intensity coercive administrative means for a “one-size-fits-all” type of pollution control, resulting in a mismatch of resources, increased costs, and decreased efficiency. In these industrial cities, economic development, technological innovation and human capital have no significant moderating effect on the relationship between environmental regulation and TFP.

Analyzing the results of Table 4, Table 5 together, it can be concluded that government intervention may be the more important and fundamental urban endowment factor, which may influence the moderating outcomes of other endowment factors. In fact, government intervention is a complex concept; when defined as undue intervention in the market, government intervention is detrimental to environmental regulatory outcomes [77]; when defined as appropriate intervention such as information sharing, financial support for R&D, and technological development assistance, government intervention helps firms to improve their technological innovation capabilities [78]. Therefore, the moderating effect of government intervention on environmental regulation is urban heterogeneous. In Beijing, appropriate means of government intervention, including the use of market incentives and guidance, can integrate the city's economic, technological and human capital advantages to form a synergy that jointly promotes and supports technological innovation by enterprises. Those industrial cities, on the other hand, mainly use coercive administrative means, which restricts the compliance options of enterprises and increases their compliance costs; while restraining and distorting market functions, it also weakens the supportive role of other urban endowment factors, thus adversely affecting the resource optimization and technological innovation capacity of enterprises.

## 5 Conclusions

This paper analyzes the moderating effect of urban endowments on the environmental regulations-productivity relationship and its heterogeneous mechanisms. We find that, as the intensity of environmental regulation increases, production in high-polluting industries in Beijing shows an “innovation compensation” effect and an “inefficient enterprise exit” effect, while in industrial cities, production in high-polluting industries shows an “innovation compensation” effect in the early stages and a “compliance cost” effect in the later stages, in contrast to the trend presented in Beijing.

We argue that these differences arise from the heterogeneous moderating effect of different levels of urban endowment factors on the environmental regulation-productivity relationship. High levels of urban endowment factors, such as a city's economic, technological and human capital, can provide positive conditions to support the productivity development of firms under environmental regulatory pressure, thereby mitigating the “compliance cost” effect and enhancing the “innovation compensation” effect. However, low levels of urban endowment factors are incapable of providing a supportive moderating role for firms under pressure from environmental regulation. The above findings imply that without supportive government strategies, the continued strengthening of environmental regulations will further widen the gap between the environmental and economic development within the region.

We also argue that government intervention may have a “double-edged sword” effect on the moderating role of urban endowments. Appropriate policy instruments can correct market failures, thus further activating the positive moderating role of urban endowment. In contrast, simple and brutal “one-size-fits-all” administrative intervention can further exacerbate market failures and reduce production efficiency. Specifically, among polluting enterprises, those in high- and medium-polluting sectors are subject to greater actual regulatory pressure and require government intervention using guidance and incentive policies such as optimizing property rights systems, tax breaks and financial subsidies to promote and support innovation; enterprises in low-polluting sectors contribute less pollution and, under the dual pressure of environmental regulation and market competition, will choose resource allocation and pollution control options that will achieve minimize costs while contributing to the advancement of social welfare. Thus, for low-pollution enterprises, reducing excessive command-based administrative intervention can often improve their economic efficiency.

The findings of this paper have important policy implications for China and other developing countries, especially those with large regional development gaps. Firstly, within the framework of the Paris Agreement to address climate change, the intensity of environmental regulation will continue to increase in countries around the world, thereby exerting pressure on economic performance, especially for countries lagging behind in development; secondly, in countries with large regional development gaps, the impact of environmental regulation on economic development will show significant heterogeneity; thirdly, developing countries tend to have less developed markets and lower regulatory capacity, and therefore tend to resort to administrative interventions in social and environmental management, which may have a more adverse impact on economic development. More research is warranted in major cities in developing countries with the above characteristics to test the robustness of our results. In addition, despite the long time span of this study (15 years), the study period had to be cut off to 2017 due to limitations in the availability of data on energy and pollutant removal rates across industrial sectors; the impact of subsequent environmental regulation and economic changes, particularly the Covid-19 epidemic, on our conclusions remains to be examined.

## Ethics statement

Not applicable.

## Data availability statement

This paper uses panel data from 2003 to 2017 for six major cities in the Beijing-Tianjin-Hebei region of China as a sample. And the data are obtained from the China City Statistical Yearbook, China Industrial Statistical Yearbook, China Environmental Statistical Yearbook, Hebei Economic Yearbook, Statistical Yearbooks, and Statistical Bulletins of each city.

## Additional information

No additional information is available for this paper.

## CRediT authorship contribution statement

**Mingyang Wang:** Writing – original draft, Methodology, Formal analysis. **Rong Gao:** Methodology, Investigation, Formal analysis. **Hua Ma:** Writing – review & editing, Supervision, Project administration, Funding acquisition, Conceptualization.

## Declaration of competing interest

The authors declare that they have no known competing financial interests or personal relationships that could have appeared to influence the work reported in this paper.
